# Formation of ZrC–SiC Composites from the Molecular Scale through the Synthesis of Multielement Polymers

**DOI:** 10.3390/ma14143901

**Published:** 2021-07-13

**Authors:** Fabien Bouzat, Romain Lucas, Yann Leconte, Sylvie Foucaud, Yves Champavier, Cristina Coelho Diogo, Florence Babonneau

**Affiliations:** 1 IRCER, Université de Limoges, UMR 7315, F-87068 Limoges, France; fbo@shimadzu.fr (F.B.); sylvie.foucaud@unilim.fr (S.F.); 2 Université Paris-Saclay, CEA, CNRS, NIMBE, 91191 Gif-sur-Yvette, France; yann.leconte@cea.fr; 3 Service de RMN, BISCEm (US042 INSERM-UMS 2015 CNRS), CBRS, 2 rue Bernard Descottes, F-87025 Limoges, France; yves.champavier@unilim.fr; 4 Institut des Matériaux de Paris-Centre, CNRS, Sorbonne Université, FR2482, F-75005 Paris, France; cristina.coelho@upmc.fr; 5 Laboratoire de Chimie de la Matière Condensée de Paris, CNRS, Sorbonne Université, LCMCP, F-75005 Paris, France; florence.babonneau@sorbonne-universite.fr

**Keywords:** zirconium carbide, silicon carbide, multielement polymer, thermal behaviour

## Abstract

In the field of non-oxide ceramic composites, and by using the polymer-derived ceramic route, understanding the relationship between the thermal behaviour of the preceramic polymers and their structure, leading to the mechanisms involved, is crucial. To investigate the role of Zr on the fabrication of ZrC–SiC composites, linear or hyperbranched polycarbosilanes and polyzirconocarbosilanes were synthesised through either “click-chemistry” or hydrosilylation reactions. Then, the thermal behaviours of these polymeric structures were considered, notably to understand the impact of Zr on the thermal path going to the composites. The inorganic materials were characterised by thermogravimetry-mass spectrometry (TG-MS), X-ray diffraction (XRD), and scanning electron microscopy (SEM). To link the macromolecular structure to the organisation involved during the ceramisation process, eight temperature domains were highlighted on the TG analyses, and a four-step mechanism was proposed for the polymers synthesised by a hydrosilylation reaction, as they displayed better ceramic yields. Globally, the introduction of Zr in the polymer had several effects on the temperature fragmentation mechanisms of the organometallic polymeric structures: (i) instead of stepwise mass losses, continuous fragment release prevailed; (ii) the stability of preceramic polymers was impacted, with relatively good ceramic yields; (iii) it modulated the chemical composition of the generated composites as it led, inter alia, to the consumption of free carbon.

## 1. Introduction

**Figure 1:**
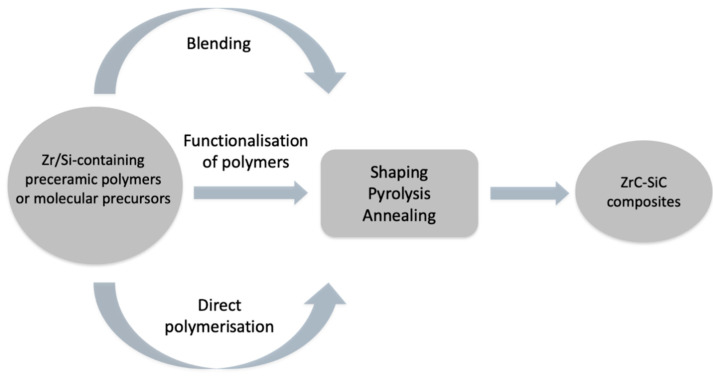
A summary of different strategies described to obtain ZrC–SiC composites using the PDCs route [9].

Advanced technologies in the nuclear and aeronautic fields require high-performance materials [1,2]. In this field, non-oxide ceramics are considered as materials of choice thanks to their good thermomechanical properties and high melting points, up to 3000 °C in the case of Ultra-High-Temperature Ceramics (UHTCs) [3,4]. In particular, ZrC is a promising material due to the combination of a high melting point (3400 °C) with interesting hardness, fracture toughness, and strength [5]. Nevertheless, ZrC resistance to oxidation must be improved when considering applications at high temperatures in oxidising environments. To solve this drawback, SiC and Si-based composites were selected. Indeed, SiC can form a protective layer of silica in specific passive oxidation conditions [6]. In parallel, the polymer-derived ceramics (PDCs) route is a promising way to fabricate homogenous materials by designing polymer networks at the molecular level, for example, using tailored polycarbosilanes (PCS) to generate SiC ceramics [7,8,9,10]. Regarding the literature, three main ways can be highlighted to incorporate a metallic element, such as group 4 metals, into polymeric structures (Figure 1) [11]. First, a simple blending of polymers with metals can lead to non-oxide precursors of ceramic composites. By changing the scale of action in terms of chemical reactivity, it is also possible to modify polymers by functionalising them with suitable key molecules and then introducing new elements into the macromolecular structure. Thanks to this strategy, polymers with pendant groups containing transition metals can be synthesised. Finally, direct polymerisation starting with molecular precursors is possible. With these three different routes in mind, it is worth mentioning that the level of uniformity of the distribution of metal ions in the polymer will condition the homogeneity of the generated ceramics/composites in terms of composition, microstructure, and phase distribution.

Via this process, polymers are transformed into ceramics through a suitable pyrolysis step that needs to be understood and, if possible, mastered. That is why the thermal behaviour needs to be studied to see the impact of the polymer structure regarding its thermal ability, the ceramic yield, and the involved pyrolytic mechanisms [12,13,14]. This methodology is useful for identifying the best structure for a polymer to lead to defined non-oxide ceramics in the best conditions. 

In this context, this study describes the impact of PCS structures on their thermal behaviour for different macromolecular structures, including zirconium-containing PCS. They were synthesised at the molecular level by using either a click-chemistry reaction, copper (I)-catalysed alkyne-azide 1,3-dipolar cycloaddition (CuAAC), [15,16,17], or a hydrosilylation polymerisation reaction [18,19]. The thermal behaviour of each polymer is investigated by trying to link the macromolecular structure to the mechanisms involved during the ceramisation process. Particularly, the structure and the composition of the starting polymers and those of the final ceramics are discussed.

## 2. Materials and Methods

### 2.1. Materials

All reactions were conducted under air atmosphere. All the solvents and chemicals were commercially available and were used as received. 1,4-diethynylbenzene (96%), Karstedt’s catalyst (platinum(0)-1,3-divinyl-1,1,3,3-tetramethyldisiloxane complex solution), anhydrous dichloromethane (≥99.8%), bis (chlorométhyl)dimethylsilane (97%), sodium azide (99%), *N*,*N*-dimethylformamide (99.8%), propargyl bromide (80 wt.% in toluene), copper bromide (98%), *N*,*N*-diisopropylethylamine (99%), 1,4-diethynylbenzene (96%), and absolute ethanol (≥99.8%) were purchased from Sigma-Aldrich (St. Quentin Fallavier, France). Diphenylsilane (97%), Bis (cyclopentadienyl) zirconium (IV) dihydride (ZrCp_2_H_2_), toluene, cyclohexane (+99%), magnesium sulfate (99.5%), ammonia (35% in water), diethylic ether (99%), and petroleum ether were obtained from Alfa-Aesar. Chloroform d_1_ (99.8%) and dimethylsulfoxide d_6_ (99.8%) were purchased from EurisoTop (Saint Aubin, France).

### 2.2. Characterisations

The characterisations by IR spectroscopy of solid samples were performed using Fourier Transform IR equipment (Spectrum One FT-IR, Perkin-Elmer, Villebon-sur-Yvette, France). These analyses were carried out in transmittance mode using KBr pellets (4000–400 cm^−1^). ^1^H and ^13^C NMR spectra were recorded at 500.15 MHz and 125.76 MHz, respectively, using a Bruker Avance III HD 500 MHz NMR spectrometer (B_0_ = 11.4 T, Palaiseau, France). Chemical shifts (δ) are expressed in parts per million with tetramethylsilane as an internal standard (δ = 0 ppm). Data are reported as follows: chemical shift, multiplicity (s, singlet; d, doublet; t, triplet; m, multiplet), coupling constants (Hz), and assignment. For solid-state NMR, on the one hand, ^13^C CPMAS NMR spectra were recorded on a Bruker Avance 700 MHz spectrometer at B_0_ = 16.4 T, ν_0_(^13^C) = 175.95 MHz using a CP-MAS Bruker probe with a 2.5 mm ZrO_2_ rotor spinning at 20 kHz. On the other hand, ^29^Si CPMAS NMR spectra were recorded on a Bruker Avance 700 MHz spectrometer at B_0_ = 16.4 T, ν_0_(^29^Si) = 139.04 MHz using a CP-MAS Bruker probe with a 4 mm ZrO_2_ rotor spinning at 5 kHz. Chemical shifts were referenced to tetramethylsilane (δ = 0 ppm). The thermal behaviour of the precursors was followed by means of differential scanning calorimetry and thermal gravimetric analysis (DSC-TG, Jupiter, STA 449 F3, Dardilly, France) in an argon atmosphere at a heating rate of 5 °C·min^−1^ (argon flow: 20 mL·min^−1^) coupled with mass spectrometry (Omnistar, Balzers Instrument, Annecy, France). Phase identification was carried out on powders using a Siemens D5000 X-ray diffractometer (Saint-Denis, France) with a θ–2θ configuration and Cu Kα radiation.

### 2.3. Synthesis

**Figure 2:**
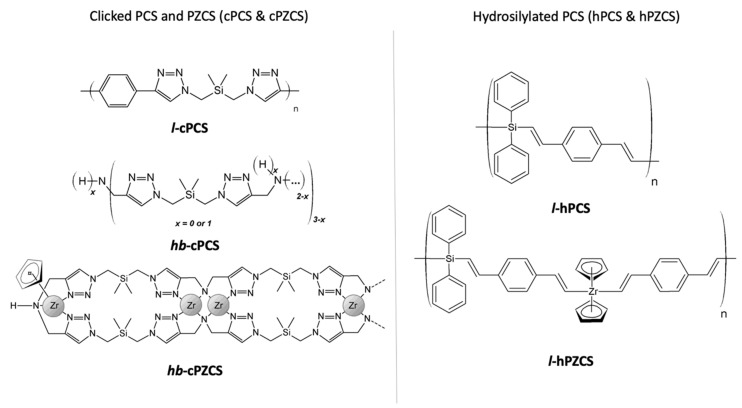
Different preceramic precursors synthesised by polycycloaddition (left) or polyhydrosilylation/polyhydrozirconation (right). To simplify the hypothetical ***hb*-cPZCS** formula, no charges or oxidation states are indicated.

For the following syntheses, the different polymer structures are summed up in Figure 2.

#### 2.3.1. Polycycloaddition (CuAAC)

##### Linear polycarbosilane (***l*-cPCS**, as *linear*-clicked PCS)

Experiments were carried out following a process described by Wang and coworkers [20]. The polymer was prepared by the reaction of bis(azidomethyl)dimethylsilane [21,22] (763 mg; 4.3 mmol) with 1,4-diethynylbenzene (540 mg; 4 mmol), 0.1 equiv of copper bromide (0.4 mmol), and 0.6 equiv of DIPEA (2.6 mmol) in DMF (5 mL). This mixture was then stirred during 24 h at 60 °C. After a treatment using water (100 mL), the light brown precipitate was filtered with a Büchner funnel, and several washes were carried out with ammonia to remove the copper. The solvent was then removed by evaporation under reduced pressure to obtain the linear polymer, a brown powder, named ***l*-cPCS** (1.42 g, 84%). ^1^H NMR (500 MHz, DMSO-d_6_, δ): 8.71–8.34 (m, CH_triazole_), 7.90–7.83 (m, CH_Ar_), 4.13-3.92 (m, Si-CH_2_), 3.81 (s, N-CH_2_), 0.27-(-)0.06 (m, Si-CH_3_); ^13^C NMR (125 MHz, DMSO-d_6_, δ): 162.3, 133, 125.6–125.3 (CH_Ar_), 122.4 (CH_triazole_), 36.4 (Si-CH_2_), 35.8 (N-CH_2_), -4.7 (Si-CH_3_); IR (KBr), σ_max_ (cm^−1^): 3148, 2960, 1641, 1432, 1253.

##### Hyperbranched polycarbosilane (***hb*-cPCS**, as *hyperbranched*-clicked PCS)

The polymer was prepared by the reaction of bis (azidomethyl) dimethylsilane [21,22] (763 mg; 4.3 mmol) with di- and tripropargylamine [23] (500 mg; 8.6 mmol), 0.1 equiv of copper bromide (0.4 mmol), and 0.6 equiv of DIPEA (2.6 mmol) in DMF (5 mL). This mixture was then stirred for a duration of 24 h at room temperature. Purification was performed via liquid-liquid extraction using water (10 mL) and diethyl ether (20 mL). The organic phase was cleaned using water (10 mL) and was then dried with MgSO_4_; the product was then filtered. Afterwards, the solvent was removed by evaporation under reduced pressure to obtain the hyperbranched polymer ***hb*-cPCS** (1.522 g, 92%). ^1^H NMR (500 MHz, DMSO-d_6_, δ): 8.30–7.58 (m, CH_triazole_), 4.61–3.82 (m, Si-CH_2_), 3.70 (br s, N-CH_2_), 0.25–0.04 (m, Si-CH_3_); IR (KBr), σ_max_ (cm^−1^): 3146, 2958, 2848, 1656, 1260.

##### Zr-modified hyperbranched polycarbosilane (***hb*-cPZCS**, as *hyperbranched*-clicked Zr-containing PCS)

A one-pot synthesis was carried out to incorporate the Zr element into the hyperbranched polymer ***hb*-cPCS** [22]. ***hb*-cPCS** was selected due to its better mass and ceramic yields. The process for this was based on the previous one. First, bis(cyclopentadienyl)zirconium(IV)dichloride (1.105 g; 3.8 mmol) was dissolved in THF (2.5 mL) with 0.1 equiv of copper bromide (0.8 mmol) and 0.6 equiv of DIPEA (4.5 mmol). Bis(azidomethyl)dimethylsilane (3.8 mmol) was added to the mixture. Immediately following this, di- and tripropargylamine were added carefully, drop by drop. The involved reaction was very exothermic and a brown gas was released. This mixture was then stirred for a duration of 24 h at room temperature. After a treatment using water (100 mL), the black precipitate was filtered with a Büchner funnel. The solvent was then removed by evaporation under reduced pressure to obtain the multielement polymer (1.34 g, 66%). ^1^H NMR (500 MHz, DMSO-d_6_, δ): 8.71 (m, CH_triazole_), 6.57–6.46 (m, CH_Ar_), 4.45–3.81 (m, Si-CH_2_), 3.65–3.52 (m, N-CH_2_), 0.17-(-)0.07 (s, Si-CH_3_); IR (KBr), σ_max_ (cm^−1^): 2953, 2825, 1621, 1439, 1400, 1254, 465.

#### 2.3.2. Hydrosilylation

##### Linear polycarbosilane obtained by hydrosilylation (***l*-hPCS**, as *linear*-hydrosilylated PCS)

The linear polycarbosilane (PCS), precursor of SiC, was synthesised in our laboratory using diphenylsilane, 1,4-diethynylbenzene, toluene, and Karstedt’s catalyst as starting reagents. First, 1.05 g 1.4-diethynylbenzene (8.323 mmol) was added into 10 mL of toluene. Then, a solution of 0.038 mL of Karstedt’s catalyst (0.1 equiv 0.0832 mmol) was prepared in 5 mL of toluene and dropped into the mixture. Finally, 1.6 mL of diphenylsilane (1 equiv; 8.323 mmol) was added, and 5 mL of toluene was introduced to obtain a complete volume of solvent of 20 mL. The reaction mixture was first stirred at 25 °C, and the temperature was increased every 10 minutes to 40 °C, then 55 °C, and held at the final temperature of 75 °C for 24 h under magnetic stirring. After the polymerisation reaction, an excess of absolute ethanol (84.98 mL) was added to precipitate the mixture and to create an azeotrope, which facilitates the evaporation of the solvent. Removal of the solvent on a rotary evaporator, at 70 °C and under vacuum, gave an orange powder. The polymer was soluble in common organic solvents. It was obtained in a 93% yield. ^1^H NMR (500 MHz, CDCl_3_, δ): 7.68–7.44 (tdd, J = 6.5 Hz, HC_ortho_), 7.5–7.26 (m, HC_meta/para_), 7.37 (s, CH_aromatic_), 7.04–6.9 (d, J = 19.3 Hz, Si-CH) and 6.88–6.75 (d, J = 19.3 Hz, = CH); ^13^C NMR (125 MHz, CDCl_3_, δ): 148.4 (Si-CH = C), 135.9 (CH_ortho_), 129.7 (CH_aromatic_), 127.3 (CH_meta/para_), 123.6 (=CH).

##### Polyzirconocarbosilane obtained by hydrosilylation (***l*-hPZCS**, as *linear*-hydrosilylated Zr-containing PCS)

As proposed by Schwartz in 1976 [24], the incorporation of zirconium into the polymer network was expected from the use of a reagent with Zr–H functions. Due to the low solubility of bis(cyclopentadienyl)zirconium(IV) dihydride (ZrCp_2_H_2_), this compound was first dissolved in toluene at 110 °C for 24 h. The linear multielement polymer (PZCS) was then synthesised by adding diphenylsilane (1 equiv) to the ZrCp_2_H_2_ solution. Separately, 1,4-diethynylbenzene (2 equiv) was dissolved in toluene in the presence of Karstedt’s catalyst (0.01 mol. equiv). The reaction mixture was first stirred at 25 °C and heated under the same conditions as previously described. Then, an excess of absolute ethanol (85 mL) was added to precipitate the mixture. Removal of the solvent with a rotary evaporator, at 70 °C and under vacuum, gave a dark brown powder. The polymer, obtained in an 87% yield, was not soluble in common organic solvents. 

### 2.4. Pyrolysis

Polymer pyrolysis was carried out in a horizontal tubular furnace under flowing argon atmosphere. Temperatures of 1400 °C and 1600 °C were selected with a heating rate of 10 °C·min^−1^, and a plateau was set at the treatment temperature for 1 h. After being cooled at 20 °C·min^−1^ to the ambient temperature, the powders were collected.

## 3. Results and Discussion

### 3.1. Characterisation of the Polymeric Precursors

To obtain original precursors for SiC and ZrC–SiC ceramics, two reactions were considered: the CuAAC and hydrosilylation reactions (Figure 2 and Table 1). Indeed, they display advantages such as high reaction rates at relatively low temperatures, solvent tolerance, and good regioselectivity, and they lead to easy-to-purify products with excellent yields. Via these two reactions, three polycarbosilanes, named ***l*-cPCS**, ***hb*-cPCS**, and ***l*-hPCS**, and two Zr-containing polycarbosilanes, ***hb*-cPZCS** and ***l*-hPZCS**, were synthesised.

#### 3.1.1. Polycarbosilanes (***l*-cPCS**, ***hb*-cPCS**, and ***l*-hPCS**)

Polycycloaddition by means of a CuAAC reaction was carried out to reach a linear polymer (***l*-cPCS**) and a hyperbranched polycarbosilane, i.e., ***hb*-PCS**. To obtain these structures, bis (azidomethyl) dimethylsilane was added either to 1,4-diethynylbenzene or to a mixture of di- and tripropargylamine [22]. The idea to compare ***l*-cPCS** with ***hb*-PCS** was motivated by the fact that hyperbranched polymers are generally characterised by leading to inorganic materials with better ceramic yields than in the case of linear polymers [12,25]. ***l*-cPCS** and ***hb*-PCS** were obtained in good yields of 84% and 92%, respectively, and they both displayed low solubility in common organic solvents (Table 1). Another polycarbosilane was also considered, ***l*-hPCS**, through a hydrosilylation reaction exclusively based on C, H, and Si elements. As the kinetics for hydrosilylation of alkynes are favoured over those for alkenes, it was decided to use commercially available diphenylsilane and 1,4-diethynylbenzene in the presence of Karstedt’s catalyst. ***l*-hPCS** was synthesised in a 93% yield as an orange powder soluble in toluene, which is a great advantage towards different processing routes leading to non-oxide ceramics (e.g., dip-coating, spray pyrolysis, etc.), with suspensions that can lead to core–shell structures, for example [26]. These three polymers were characterised by ^1^H, ^13^C liquid-state NMR spectroscopy, confirming their structures (cf. experimental part). ^29^Si solid-state NMR was also carried out and revealed SiC_4_ environments (Si(CH_3_)_2_(CH_2_)_2_ in Figures S1–S3, or Si(C_sp2_)_4_ in Figure S4). Looking at the different polycarbosilane structures to lay the foundation for the thermal behaviour explanations, ***l*-cPCS** and ***l*-hPCS** display phenyl groups in the backbone of the polymers or as pendant groups, while ***hb*-cPCS** has a hyperbranched structure with triazole functional groups in its backbone, like ***l*-cPCS**. In addition, conjugated systems are more present in ***l*-cPCS** and ***l*-hPCS**, as triazole and phenyl are directly linked to other systems (i.e., alkenes or phenyl groups). 

#### 3.1.2. Zr-Containing Polycarbosilanes (***hb*-cPZCS**, ***l*-hPZCS**)

**Figure 3:**
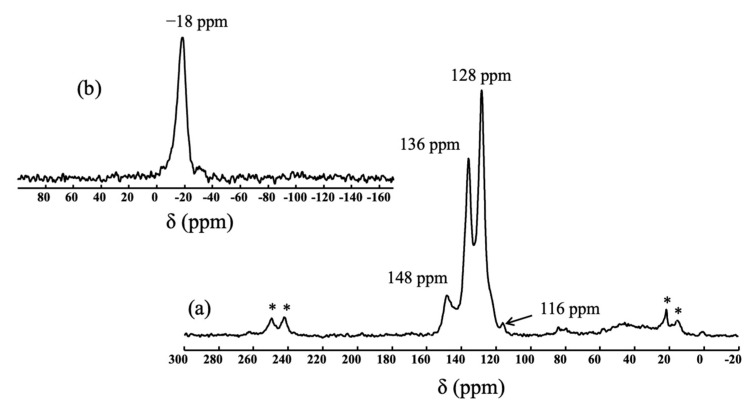
(**a**): ^13^C CPMAS NMR spectrum of ***l*-hPZCS**; * indicates rotation bands; (**b**): ^29^Si CP MAS NMR spectrum of ***l*-hPZCS**.

According to the monomers used, and depending on the reactions involved, it is possible to incorporate Zr into the polycarbosilane structures either by using the reactivity of nitrogen elements (***hb*-cPZCS**) with a zirconium complex (ZrCp_2_Cl_2_) or by employing a combined hydrosilylation/hydrozirconation reaction (***l*-hPZCS**) using ZrCp_2_H_2_. ***hb*-cPZCS** was obtained as a black solid with a satisfying yield of 66%, and it showed low solubility in common organic solvents [22]. In parallel, ***l*-hPZCS**, a brown powder, was synthesised in a better yield of 87%. It was insoluble in common organic solvents. Thus, a solid-state NMR study was carried out to elucidate the structure of the polyzirconocarbosilane. Regarding the ^13^C NMR spectrum in Figure 3a, the presence of a peak for the chemical shift of 116 ppm was attributed to the carbon atoms of the cyclopentadienyl groups bonded to Zr. Thanks to this result, it was assumed that Zr was incorporated into the polymer structure. In addition, signals at 148 ppm, 136 ppm, and 128 ppm were observed, as in the case of ***l*-hPCS**. The complementary ^29^Si NMR analysis in Figure 3b indicates that the Si environments remained the same as those for ***l*-hPCS** (signal at −18 ppm for SiC_4_). Thanks to this investigation, a structure was suggested for ***l*-hPZCS**, resulting in statistical copolymerisation of ZrCp_2_H_2_ and diphenylsilane with 1,4-diethynylbenzene (Figure 2). 

**Table 1:** Characteristics of the different synthesised polymers and their respective yields.

Polymer	Reaction	Aspect	Solubility in Toluene *	Polymer Yield (%)
***l*-cPCS**	Click-chemistry (CuAAC)	Brown solid	-	84
***hb*-cPCS**	Click-chemistry (CuAAC)	Orange gel	-	92
***hb*-cPZCS**	Click-chemistry (CuAAC)	Black solid	-	66
***l*-hPCS**	Hydrosilylation	Orange solid	++	93
***l*-hPZCS**	Hydrosilylation & Hydrozirconation	Brown solid	--	87

* (-) low solubility, (--) insoluble, (++) very soluble.

Following the synthesis of the different polymers with satisfying yields, displaying linear or hyperbranched structures and incorporating zirconium or not, it was decided to investigate their thermal behaviour in order to determine the impact of the composition/structure on the ceramic yield and on the nature of the compounds released during thermal treatment.

### 3.2. Thermal Behaviour of the Preceramic Polymers

#### 3.2.1. Polycarbosilanes

##### PCS Obtained through CuAAC: ***l*-cPCS** and ***hb*-cPCS**

**Figure 4:**
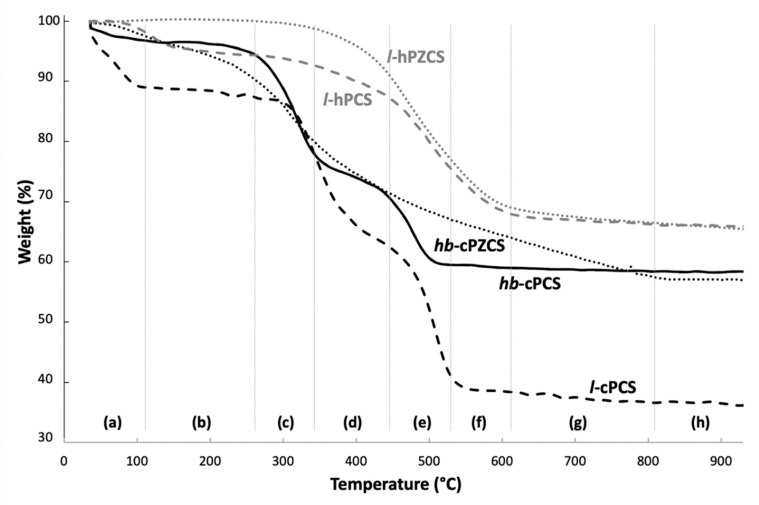
TG profiles from thermal analysis of the different preceramic precursor PCS (**a**–**h**: temperature domains).

Figure 4 presents the thermogravimetric analysis curves of the two polycarbosilanes ***l*-cPCS** and ***hb*-cPCS** (see patterns in Figure 2). The two samples have quite similar behaviours. From eight temperature domains, five main steps are highlighted on the thermograms of these polymers ((a), (b), (c), (d) + (e), (f) + (g) + (h)). The total mass loss is higher for ***l*-cPCS**. Zone (a), for temperatures below 100 °C, reveals for ***l*-cPCS** a weight loss of 11%, which can be explained by the evaporation of water physisorbed or trapped in the polymer. This mass loss is less important (about 3%) for the ***hb*-cPCS** sample. This release of water reflects the difficulties encountered in completely purifying the precursors. Zone (b) corresponds to a temperature range of between 100 and 300 °C, for which the samples are thermally stable. Beyond 300 °C, zones (c) + (d) appear. Over this temperature range, the mass change observed for each sample is in the order of 25%. This can be attributed to evaporation of the oligomers and to the onset of degradation of the polymers. For each of the polymers, it is conceivable that C–N and Si–C bonds broke first (Table S1). In fact, above 300 °C, Si–CH_3_ bonds are broken and CH_3_ groups, volatilised, can fragment and recombine in the form of methane [14,26,27,28]. It should be noted that in the case of the polymer ***hb*-cPCS**, the mass loss started 55 °C before that observed for ***l*-cPCS**. This temperature shift can be linked to the structure of the macromolecular network, which influences the thermal stability of the polymers. In the case of ***l*-cPCS**, the unit presented in Figure 2 is highly conjugated. The presence of the aromatic ring can thus delay the decomposition of the polymer. In this temperature range, it is possible to envisage that the polymers are fragmented into two segments, one rich in C and N, and the other composed of Si and C. These two fragments then undergo modifications linked to the increase in temperature. Indeed, an additional weight loss is noted in range (e) between 440 and 555 °C. It is in the order of 30% for the sample ***l*-cPCS** and about 16% for ***hb*-cPCS**. These mass losses can be attributed to the dissociation of the triazole bonds (for ***l*-cPCS** and ***hb*-cPCS**) and of the aromatic ring (for ***l*-cPCS**). At this stage of pyrolysis, it is likely that an amorphous carbon phase will begin to form. Finally, during zones (f) + (g) + (h), the samples do not lose mass, showing thermal stability above 555 °C. The polymer-to-ceramic conversion can take place without mass loss [7,29]. The ceramic yield of the linear polymer ***l*-cPCS** is 36%, while the precursor ***hb*-cPCS**, of hyperbranched structure, has a ceramic yield of 58%. This result is consistent with the data in the literature, since hyperbranched precursors are generally associated with better ceramic yields than linear polymers [25]. The DSC curves of the two polymers have similar shapes (Figures S5 and S6). A major exothermic event coincides with the main mass change recorded in TGA and the simultaneous creation of chemical bonds. This exothermic signal is observed at roughly 511 °C for ***l*-cPCS** and at 489 °C for ***hb*-cPCS**. This phenomenon marks the organic–inorganic transition. Due to its highly crosslinked polymer network, the polymer ***hb*-cPCS** tends to organise itself at a lower temperature than the polymer ***l*-cPCS** [30]. This step would result in the formation of amorphous SiC, while at a higher temperature, SiC is able to crystallise. A hyperbranched architecture therefore makes it possible to increase the ceramic yield of the polymer (+22%). 

After pyrolysis at 1400 °C of the polymers ***l*-cPCS** and ***hb*-cPCS**, the predominant phases present are β-SiC and graphitic carbon. The presence of Cu_x_Si_1−x_-type impurities is also noted, which testifies to the difficulties in purifying the precursor during the syntheses. From the elementary analyses carried out on the pyrolysed products, the free carbon content is 12.3 wt.% for the pyrolysis residue of ***l*-cPCS** and 18.4 wt.% for that of ***hb*-cPCS**. 

In addition, the pyrolysis residues of ***l*-cPCS** and ***hb*-cPCS** are contaminated with nitrogen (between 1.7 and 2.7 wt.%). This is explained by the implementation of click-chemistry reactions. While nitrogen is a sought-after element for generating triazole bonds, which offer excellent chemical stability to polymers and facilitate synthesis conditions, a minor amount remains in the final powders. However, the absence of nitrided phases in pyrolyzed materials is explained by the absence of Si–N bonds in the polymer motif and supports the hypothesis that triazole bonds mainly decompose into non-reacting N_2(g)_ during the ceramisation step. Due to the difficulties in controlling the chemical composition of the precursors resulting from the synthesis by click-chemistry (***l*-cPCS** and ***hb*-cPCS**), the synthetic route by hydrosilylation was undertaken and led to the development of a preceramic polymer with a linear architecture, ***l*-hPCS**.

##### Two Linear Preceramic Precursors: ***l*-cPCS** and ***l*-hPCS**

The thermal behaviours of the two linear polymers ***l*-cPCS** and ***l*-hPCS** (Figure 4) were compared (Figure 2). The thermograms of these two compounds show clearly different patterns. In the case of ***l*-hPCS**, the weight loss reflecting the decomposition of the precursor takes place in a single step between 360 and 640 °C, whereas for ***l*-cPCS**, this mass change occurs at a lower temperature, between 300 and 550 °C, with two successive mass losses. This difference can be explained by the initial structure of the polymers. Indeed, the presence of C–N bonds in the backbone of ***l*-cPCS**, identified as the weakest bonds (Table S1), increases the kinetics of the polymer decomposition. For ***l*-hPCS**, the DTA signal indicates an exothermic phenomenon at around 780 °C (Figure S7), which can be attributed to the start of the polymer-to-ceramic transformation, while this phenomenon occurs at a lower temperature (i.e., 511 °C) for ***l*-cPCS**. It should also be noted that the ceramic yield of ***l*-hPCS** is 62%, much higher than that of ***l*-cPCS** (36%). The chemical composition of ceramics resulting from the pyrolysis of ***l*-cPCS** and **l-hPCS** consists mainly of the β-SiC phase and residual carbon [22,30]. 

#### 3.2.2. Zr-Containing Polycarbosilanes (PZCS)

##### PCS and PZCS Obtained through Click-Chemistry: ***hb*-cPCS** and ***hb*-cPZCS**

The study of ***hb*-cPZCS** involved observing the thermal behaviour of the ZrCp_2_Cl_2_ monomer, which was the carrier reagent for the Zr element. The thermogram of this compound indicates that ZrCp_2_Cl_2_ volatilises from 235 °C (Figure S8). Above 385 °C, the remaining residue (6%) is zirconia, resulting from an oxidation reaction between the monomer and alumina in the crucible. In the same test carried out in a pyrolysis chamber with a glassy carbon crucible, the monomer decomposed in its entirety. In addition, polymers ***hb*-cPCS** and ***hb*-cPZCS** are the closest in terms of their structures (Figure 2), as the only difference is the use of ZrCp_2_Cl_2_ for ***hb*-cPZCS**. For the latter, the mass loss is continuous, and DSC analysis can distinguish a major exothermic phenomenon at 407 °C (Figure S9). However, the thermal behaviour of ***hb*-cPZCS** is different from that of ***hb*-cPCS**, as mass losses for ***hb*-cPCS** are stepwise, while mass loss for ***hb*-cPZCS** is continuous. Therefore, the complexation of Zr ions by nitrogen ligands seems to influence the temperature fragmentation mechanisms of organometallic polymeric structures.

Focusing on the different stages of the TG analyses for these two clicked polycarbosilanes, the first part deals with the evaporation of the solvent residues (water and THF, zone (a)). Then, the signal on the DSC curve between 235 and 335 °C can be associated with the volatilisation of oligomers and ZrCp_2_Cl_2_ residues, the decomposition of which is observed in this temperature range, as revealed by the TG curve (Figures S8 and S9). Polymer decomposition also started in this temperature range. Overall, the onset of weight loss occurs at a lower temperature for ***hb*-cPZCS** than what is observed for the decomposition of ***hb*-cPCS**. Indeed, the latter presents thermal stability in zone (b), unlike ***hb*-cPZCS**. This different behaviour may be linked to the presence of Zr in the precursor backbone that can weaken C–N bonds. For the two polymers, volatilisation of the cross groups of the polymer takes place at the same time, by the breaking of Si–C bonds (Table S1). It is also possible that Zr–N bonds are weakened under the effect of temperature. The organic–inorganic transition also seems to have shifted towards the lowest temperatures for ***hb*-cPZCS**. Indeed, the exothermic peak detected by DSC analysis, which may correspond to the transformation of the polymer into an amorphous material, is located around 407 °C for ***hb*-cPZCS**, versus 489 °C for ***hb*-cPCS**. The polymer network created by complexation therefore seems to facilitate the ceramisation step. Above 800 °C (zone (h)), ***hb*-cPZCS** is thermally stable and the ceramic yield reaches a value of 54%, slightly less than that for ***hb*-cPCS** (58%). Referring to the DSC analysis curves, it can be concluded that the more hyperbranched the architecture of the precursor, the more rapid the transition to an amorphous material. It is therefore possible to modulate the composition and thermal behaviour of the precursors depending on the monomers selected.

##### PCS and PZCS Obtained through Hydrosilylation: ***l*-hPCS** and ***l*-hPZCS**

The TG curve of ***l*-hPCS** indicates four different stages, starting with zone (a), that concern the release of residual toluene detected by MS analysis. This phenomenon matched with the boiling point of this solvent (Figure 4 and Figure S10). In zone (b), no significant mass loss was observed. However, crosslinking certainly occurred with temperature. Then, from zones (c) to (f), the release of phenyl groups from diphenylsilane was detected by MS, involving a second mass loss (Figure 4, Figures S10 and S11). Finally, zones (g) and (h) did not reveal any mass loss, indicating the thermal stability of the product. In addition, the first derivative of the DTA curve showed a broad exothermic peak centred at roughly 800 °C, probably due to the conversion of the material from an organic PCS to an amorphous SiC and its structuring (Figure S11). Another endothermic peak at 1390 °C could reveal a reaction between the Al_2_O_3_ crucible and SiC. According to the TG curve, the ceramic yield of the linear polymer was about 62%. 

Through a study performed under the same conditions as for ***l*-hPZCS**, it was possible to determine the influence of zirconium on the thermal behaviour of these preceramic precursors. The thermal behaviour recorded for the monomer ZrCp_2_H_2_ (Figure S12) was characterised by a continuous mass loss from 100 °C to 1200 °C leading to the formation of a carbon-rich and Zr-containing residue. This thermal behaviour is clearly distinguishable from that of the multielement polymer ***l*-hPZCS** (Figure 4), characterised by a plateau (i.e., stable mass) from room temperature to 320 °C and for temperatures above 650 °C, strongly suggesting the incorporation of the Zr element in the polymeric structure to ensure the thermal stability of the pyrolyzed product above 650 °C. In addition, since ZrCp_2_H_2_ constituted one-third of the monomers used in the polymerisation process, the ceramic yield of ***l*-hPZCS** should have been affected by the presence of this complex. Indeed, when used alone, ZrCp_2_H_2_ lost 70% of its mass, with mass loss even above 900 °C, which was not the case for ***l*-hPZCS** (cf. plateau in zone (h)).

A closer inspection of the TG profile of ***l*-hPZCS** showed three main steps. Firstly, the material was stable at the lowest temperatures, unlike PCS (zones (a) to (b), Figure 4). Then, residual solvent evaporation was detected by mass spectrometry analysis performed during the pyrolysis (Figure S10). It showed a low-intensity signal, in good agreement with the very low mass loss detected by TGA. Then, main mass loss started in zone (d) due to volatilisation of the aromatic cycles, as revealed by the detection of the ionised fragments due to phenyl groups. At higher temperatures, from zone (d) to (g), fragments composed of the atoms C and H were detected (e.g., CH_4_, Figure S10). Meanwhile, organic-to-inorganic transformation occurred. Indeed, the DTA analysis revealed an exothermic signal centred at 770 °C. At higher temperatures, a second exothermic signal centred at 1070 °C could indicate the crystallisation of a first phase of the composite ceramic. The last exothermic signal of low amplitude at 1325 °C could be related to the crystallisation of the second phase of the composite (Figure S11). Other endothermic signals were related to solid–solid interaction between the material and the crucible (Al_2_O_3_), consisting of local small oxidation phenomena since no mass variations were observed. 

##### Ceramisation Mechanisms

**Figure 5:**
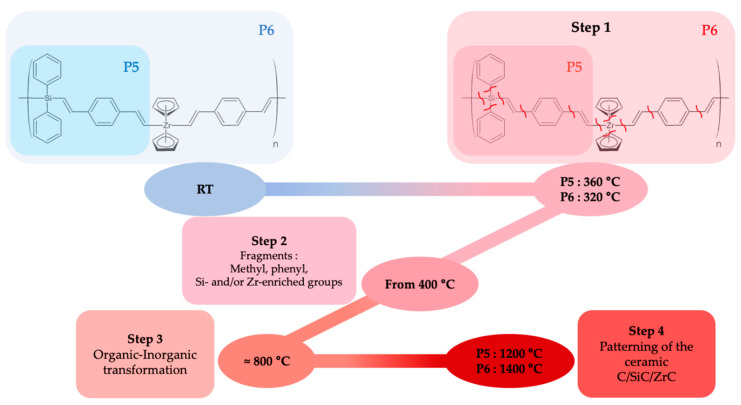
Schematic representation of the ceramisation mechanism of ***l*-hPCS** and ***l*-hPZCS**.

As ***l*-hPCS** and ***l*-hPZCS** displayed better ceramic yields, and because they only had Si, C, and H elements in their structures, they were selected to further study the mechanisms of ceramisation involved during the thermal treatment. The thermal behaviour of these two precursors could be described, first, by an analysis coupling the data collected from thermogravimetric analysis and differential thermal analysis, and by the detection of volatilised compounds during the rise in temperature by mass spectrometry. According to the previous thermogravimetric description, and to support our reasoning, a reaction mechanism in temperature was proposed (Figure 5). This mechanism can be described in four different steps: (1) breaking of the weakest bonds, just above 300 °C (start of the loss in mass, zones (a) to (c), Figure 4); (2) volatilisation or recombination of the fragments (domains (d) + (e) + (f) in Figure 4, and from mass spectrometry analyses, Figure S13); (3) molecular recombination leading to an organic-to-inorganic transformation (zone (g), Figure 4); and (4) the atomic arrangement and the patterning of the ceramics, without any mass variations (zone (h), Figure 4).

### 3.3. Ceramics Issued from **l-hPCS** and **l-hPZCS**

**Figure 6:**
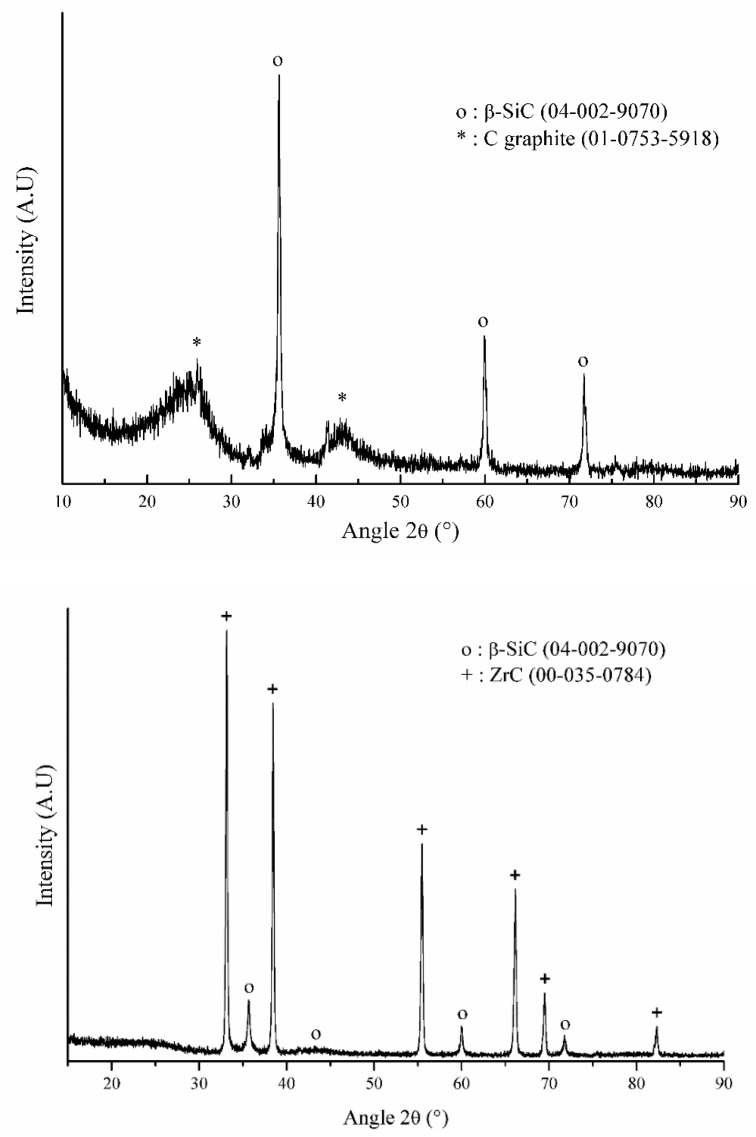
XRD patterns of polymer-derived ceramics from ***l*-hPCS** (**top**) and ***l*-hPZCS** (**bottom**).

To complete our understanding of the conversion mechanism of ***l*-hPCS** into ceramics, the precursors were pyrolysed in Ar atmosphere at 1400 °C. The obtained powders were re-annealed at 1600 °C to improve the crystallisation of the derived ceramics. The corresponding XRD patterns are shown in Figure 6. As we previously reported [30], ***l*-hPCS** led to the formation of β-SiC nanograins, as expected, dispersed in an excess of carbon graphite matrix. If we now focus the discussion on the effect of zirconium, the XRD pattern of ***l*-hPZCS**-derived ceramics showed that the final material was a ZrC/SiC composite. Semi-quantitative analysis done via the Rietveld method allowed us to estimate the composition of the crystallised phase at about 61 wt.% of ZrC and 39 wt.% of SiC. The free carbon amount was notably reduced, as no graphite was detected from the XRD pattern. Therefore, the presence of zirconium in the preceramic polymer seemed to consume free carbon to lead to ZrC at high temperatures. 

**Figure 7:**
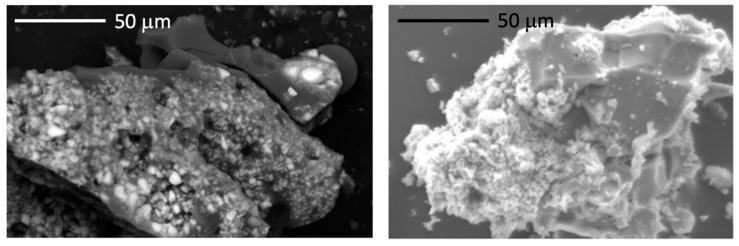
SEM image of the composite obtained from the pyrolysis of ***l*-PZCS** (**left**) compared to that of a composite generated by the pyrolysis of a commercial polymer (PCMS) mixed with ZrC powders (**right**) [31].

Concerning the distribution of the two phases, SEM images of the milled powder showed that the material was quite different (Figure 7, left). Indeed, thanks to the chemical contrast, the brightest parts of the materials were Zr-enriched phases, while the darkest were the Si-containing phases. By comparing this microstructure to a ZrC/SiC composite obtained using a commercial polycarbomethylsilane (PCMS) mixed with ZrC powders (Figure 7, right), the benefit of inserting the transition metal in the polymer through organometallic chemistry is obvious. Indeed, instead of having ZrC particles covering SiC (Figure 7, right), they seem to be embedded in a SiC matrix when using the PDC route. In addition, better ceramic yields were obtained using multielement polymers as compared to PCMS (only 26% when no crosslinking steps were carried out under oxygen).

### 3.4. Overview of the Thermal Behaviour of the Different Preceramic Polymers

The previous results have shown the possibility of synthesising composites in Si/C and Si/Zr/C systems from different organometallic precursors. The study of their thermal behaviour highlighted the relationships that link the composition and architecture of the polymers to the final ceramic materials obtained after pyrolysis. Table 2 summarises the main results obtained (ceramic yield, organic-to-inorganic transition temperature, chemical composition of the pyrolysed material, etc.) depending on the starting polymer.

On the one hand, the study of the polymers resulting from click-chemistry revealed that the ceramic yields for the hyperbranched polymers ***hb*-cPCS** and ***hb*-cPZCS**—54% and 58%, respectively—were much higher than that for polycarbosilane ***l*-cPCS**, with a linear architecture (22%). On the other hand, the hydrosilylation reaction allowed us to develop two precursors ***l*-hPCS** and ***l*-hPZCS**, which had the highest ceramic yields, around 62%. It can also be noted that the polymers resulting from click-chemistry (CuAAC), which exhibited poor solubility due to significant polymer branching, are characterised by an organic-to-inorganic transition temperature lower than those for ***l*-hPCS** and ***l*-hPZCS.** In addition, the incorporation of zirconium influenced the thermal behaviour of polymers ***hb*-cPZCS** and ***l*-hPZCS**, which exhibited continuous mass loss instead of the stepwise mass losses from the Zr-free polymers. Finally, zirconium’s benefits are twofold: it causes changes in the stability of polymers, with good ceramic yields, and it also has an impact on the chemical composition of the generated composites, as it allows for the consumption of free carbon.

## 4. Conclusions

To clarify the effect of zirconium on the formation of ZrC–SiC composites from preceramic polymers, linear and hyperbranched Zr-containing polycarbosilanes were synthesised through either CuAAC or hydrosilylation reactions. The thermal behaviours of the metal-containing polymers were investigated in comparison with those of the corresponding Zr-free polycarbosilanes. Ceramic yields in the range of 36–62% were obtained, with better results for the Zr-containing hyperbranched structures. The impact of the macromolecular structure on the ceramisation process was also considered. Introducing Zr into the structure changed the thermal behaviour from a step-by-step degradation trend for Zr-free PCSs towards continuous fragment release of species up to 900 °C. A four-step mechanism was also proposed for the hydrosilylated polymers to explain the evolution of mass changes during the thermal treatment. This approach showed two main advantages for the presence of Zr in the polymer structure: the stability of the preceramic polymers and their thermal behaviour were impacted, with relatively good ceramic yields, and it led to the consumption of free carbon. 

## Figures and Tables

**Table 2:** Some characteristics of the preceramic polymers regarding their thermal behaviour and ceramisation.

Polymer	Synthetic Process	Ceramic Yield (wt.%)	Organic-to-Inorganic Transition Range of Temperature (°C)	Ceramic Composition
***l*-cPCS**	Click-chemistry (CuAAC)	36	511	SiC + C_graphite_
***hb*-cPCS**	Click-chemistry (CuAAC)	58	489	SiC + C_graphite_
***hb*-cPZCS**	Click-chemistry (CuAAC)	54	407	SiC + ZrC
***l*-hPCS**	Hydrosilylation	62	780	SiC + C_amorphous_
***l*-hPZCS**	Hydrosilylation and Hydrozirconation	62	770	SiC + ZrC

## Data Availability

Not applicable.

## Supplementary Materials

The following are available online at https://www.mdpi.com/article/10.3390/ma14143901/s1, Figure S1: ^29^Si CP-MAS NMR spectrum of ***l*-cPCS**, Figure S2: ^29^Si CP-MAS NMR spectrum of ***hb*-cPCS**, Figure S3: ^29^Si CP-MAS NMR spectrum of ***hb*-cPZCS**, Figure S4: ^29^Si CP-MAS NMR spectrum of ***l*-hPCS**, Figure S5: DSC of ***l*-cPCS**, Figure S6: DSC of ***hb*-cPCS**, Figure S7: TG profiles of ***l*-cPCS** and ***l*-hPCS** and DATD of ***l*-hPCS**, Figure S8: TG profile of ZrCp_2_Cl_2_, Figure S9: TG profiles of ***hb*-cPCS** and ***hb*-cPZCS** and DSC of ***hb*-cPZCS**, Figure S10: Mass spectrometry of the gaseous species released during the pyrolysis of ***l*-hPCS**, Figure S11: TG profiles of ***l*-hPCS** and ***l*-hPZCS** and DATD of ***hb*-cPZCS**, Figure S12: TG profile of ZrCp_2_H_2_, Figure S13: Mass spectrometry of the gaseous species released during the pyrolysis of ***l*-hPZCS**, Table S1: Average bond enthalpy at 298 K.
