# Supplementary material for: Formation of ZrC–SiC Composites from the Molecular Scale through the Synthesis of Multielement Polymers
Source: Materials (Basel). 2021 Jul 13;14(14):3901. doi: 10.3390/ma14143901 (PMC8306986; doi:10.3390/ma14143901)
Supplement: Supplementary file 1 [file materials-14-03901-s001.zip › materials-1274791-supplementary.pdf]

materials

MDPI

Supplementary Materials

Formation of ZrC–SiC Composites from the Molecular Scale through the Synthesis of Multielement Polymers

Fabien Bouzat [1], Romain Lucas [1,*], Yann Leconte [2], Sylvie Foucaud [1], Yves Champavier [3], Cristina Coelho Diogo [4], and Florence Babonneau [5]

1 IRCER, Université de Limoges, UMR 7315, F-87068 Limoges, France; fbo@shimadzu.fr (F.B.: Fabien Bouzat); sylvie.foucaud@unilim.fr (S.F.)
2 NIMBE, CNRS, CEA, Université Paris-Saclay, 91191 Gif-sur-Yvette, France; yann.leconte@cea.fr
3 Service de RMN, BISCEm (US042 INSERM - UMS 2015 CNRS), CBRS, 2 rue Bernard Descottes, F-87025 Limoges, France ; yves.champavier@unilim.fr
4 Institut des Matériaux de Paris-Centre, CNRS, Sorbonne Université, FR2482, F-75005 Paris, France ; cristina.coelho@upmc.fr
5 Laboratoire de Chimie de la Matière Condensée de Paris, CNRS, Sorbonne Université, LCMCP, F-75005 Paris, France ; florence.babonneau@sorbonne-universite.fr (F.B.: Florence Babonneau)
* Correspondence: romain.lucas@unilim.fr; Tel.: +33587502350

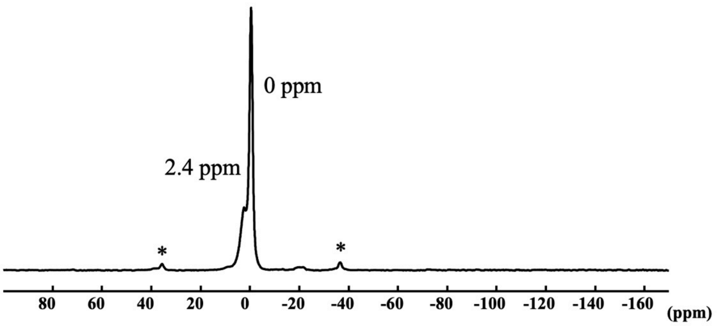

Figure S1. ^{29}Si CP-MAS NMR spectrum of ***l*-cPCS**.

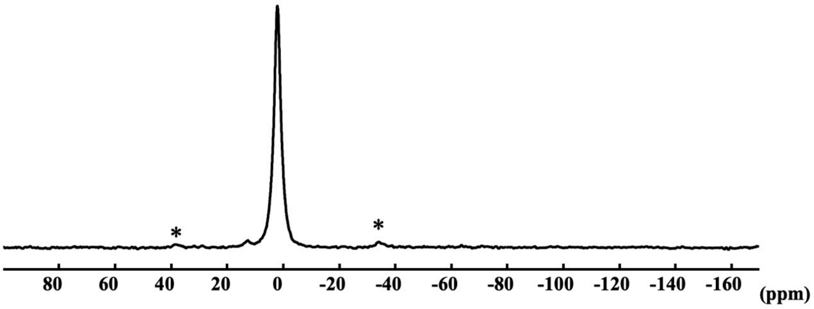

Figure S2. ^{29}Si CP-MAS NMR spectrum of ***hb*-cPCS**.

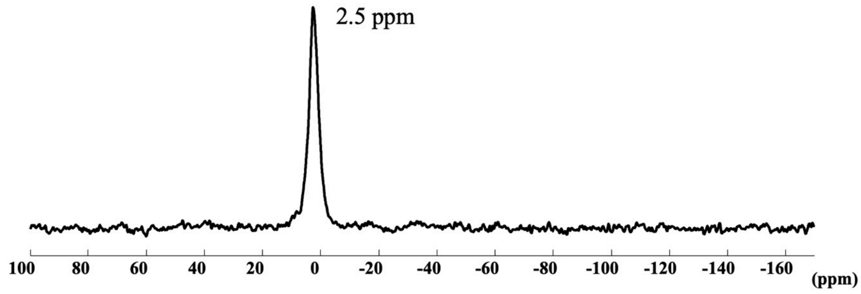

Figure S3. ^{29}Si CP-MAS NMR spectrum of ***hb*-cPZCS**.

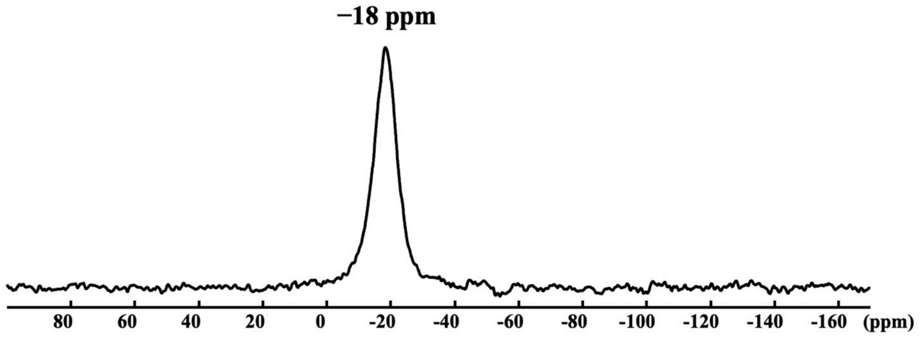

Figure S4. ^{29}Si CP-MAS NMR spectrum of ***l*-hPCS**.

Table S1. Average bond enthalpy at 298 K.

**Bond**	**Average bond enthalpy (kJ.mol^{-1})**
N–N	159
Si–C	289
C–N	293
C–C	347
N=N	418
C=C	615

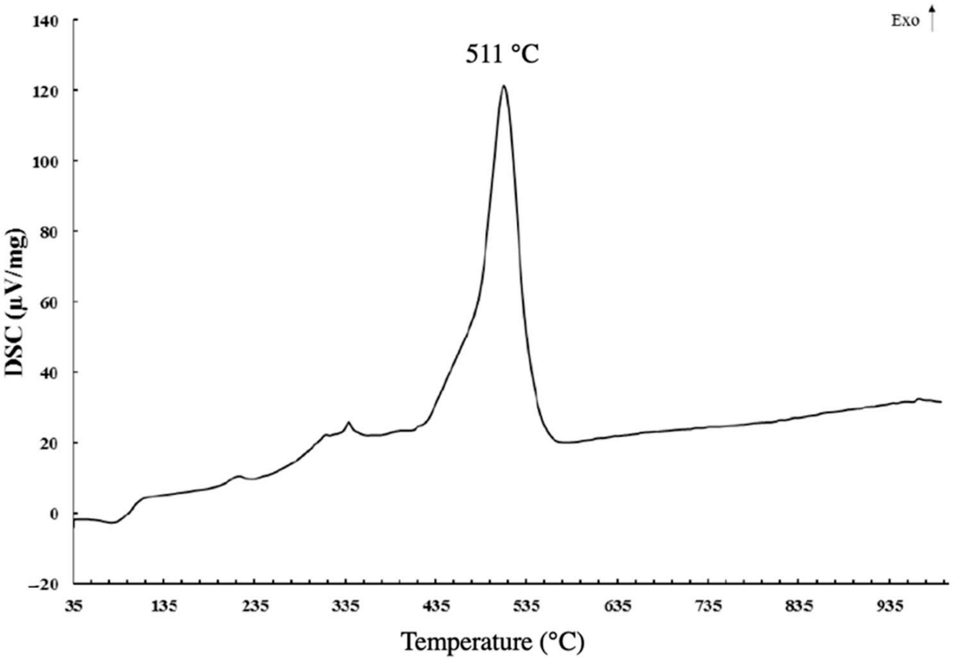

Figure S5. DSC of ***l*-cPCS** (Exo = exothermic)

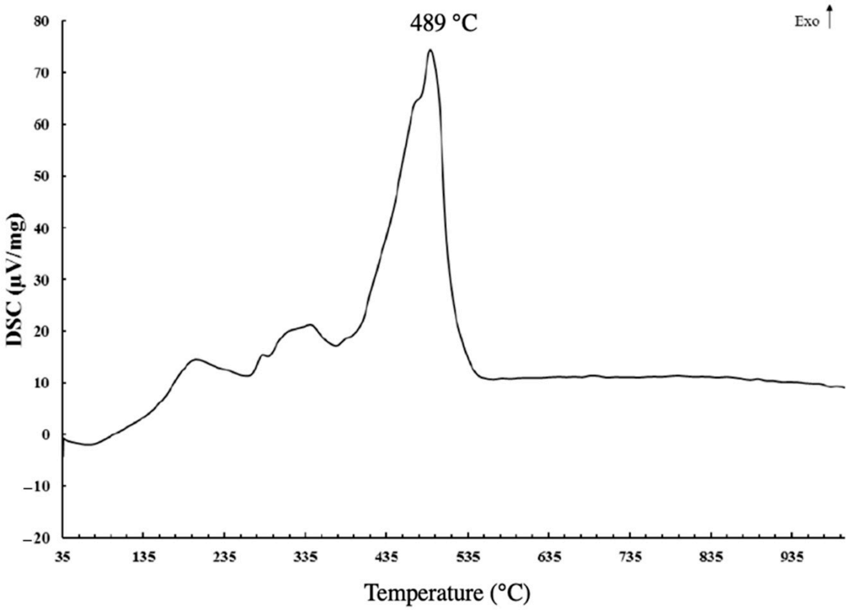

Figure S6. DSC of ***hb*-cPCS** (Exo = exothermic).

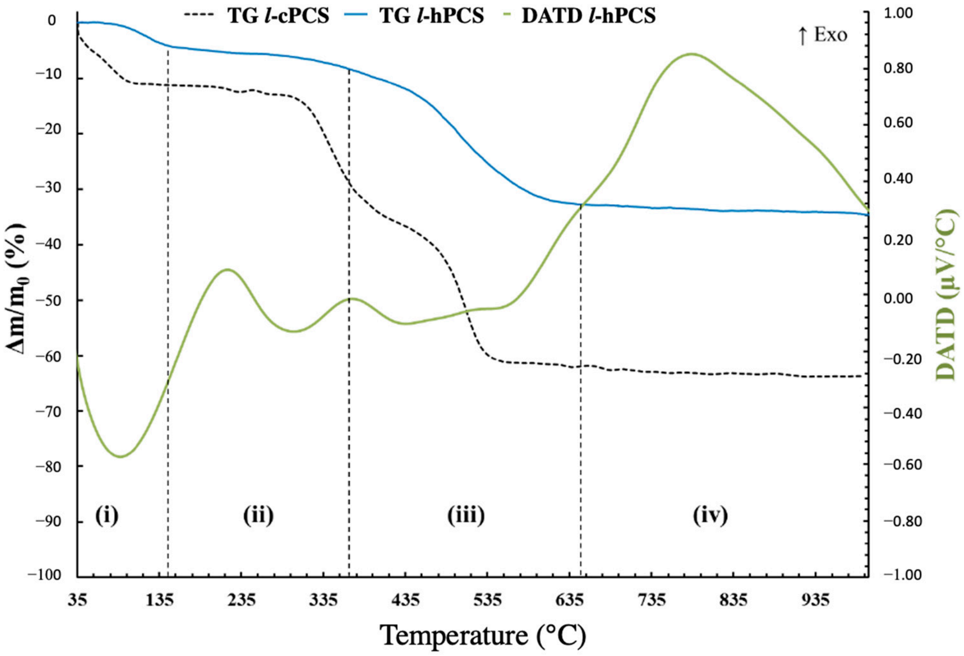

Figure S7. TG profiles of ***l*-cPCS** and ***l*-hPCS** and DATD of ***l*-hPCS**.

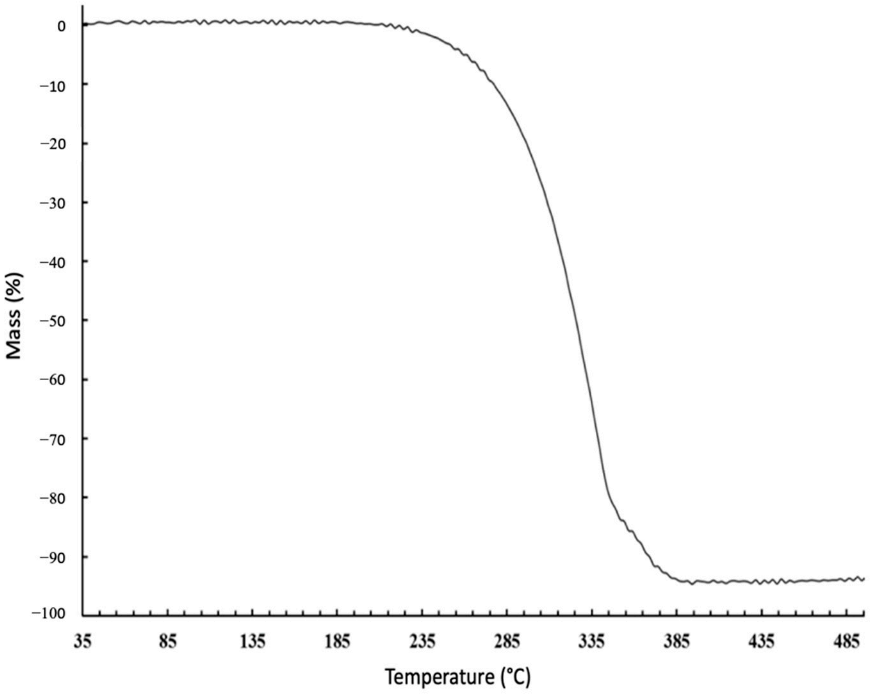

Figure S8. TG profile of $ZrCp_2Cl_2$.

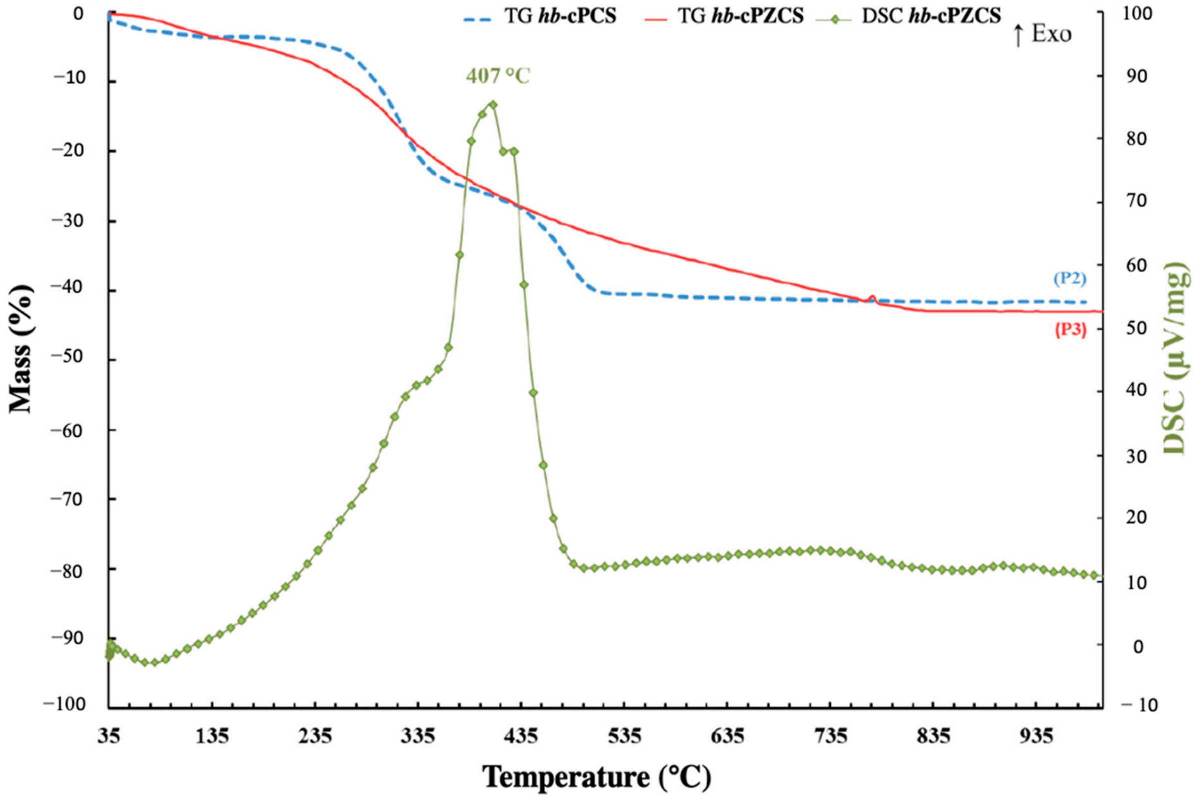

Figure S9. TG profiles of ***hb*-cPCS** and ***hb*-cPZCS** and DSC of ***hb*-cPZCS** (Exo = exothermic).

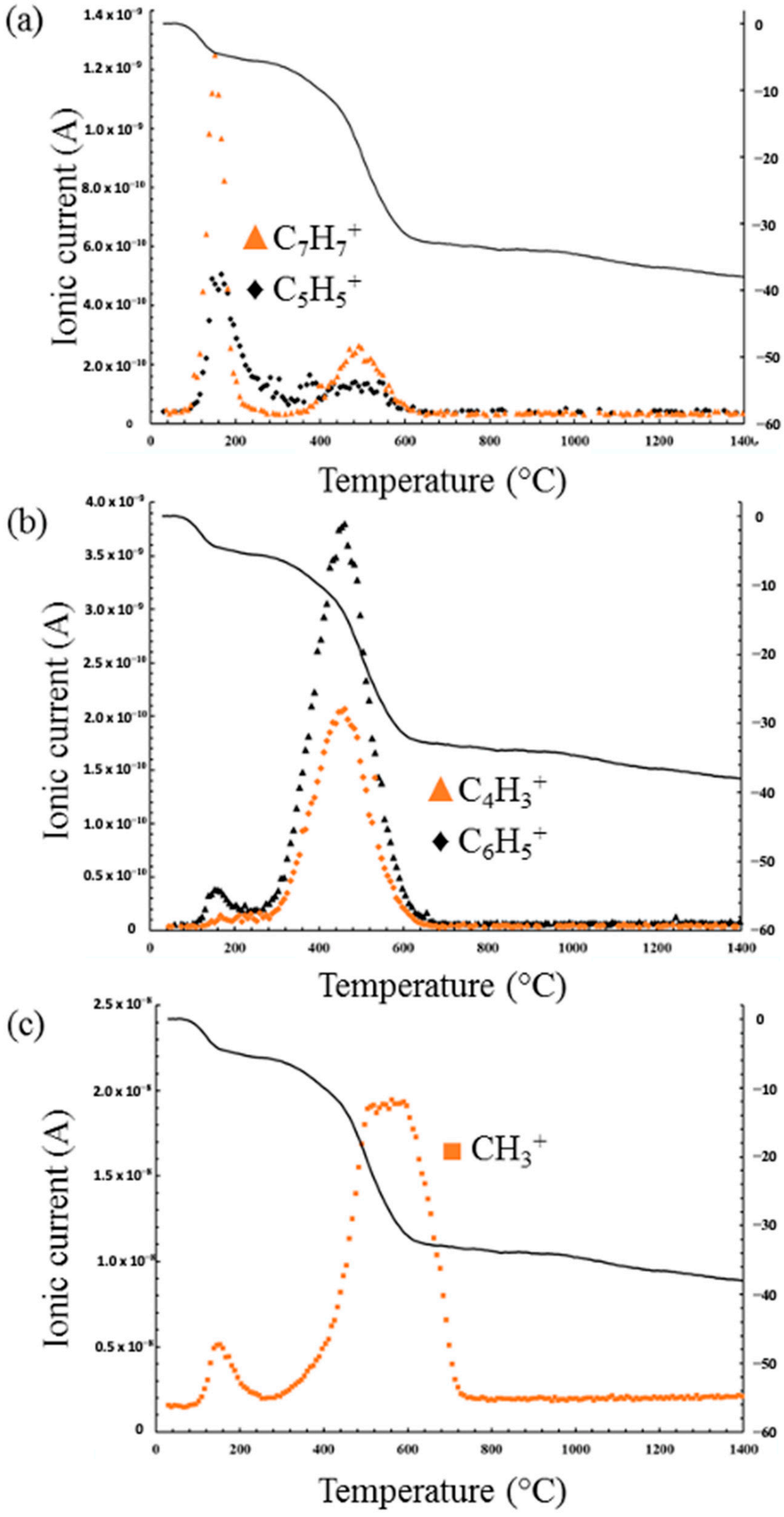

Figure S10. Mass spectrometry of the gaseous species released during the pyrolysis of ***l*-hPCS**.

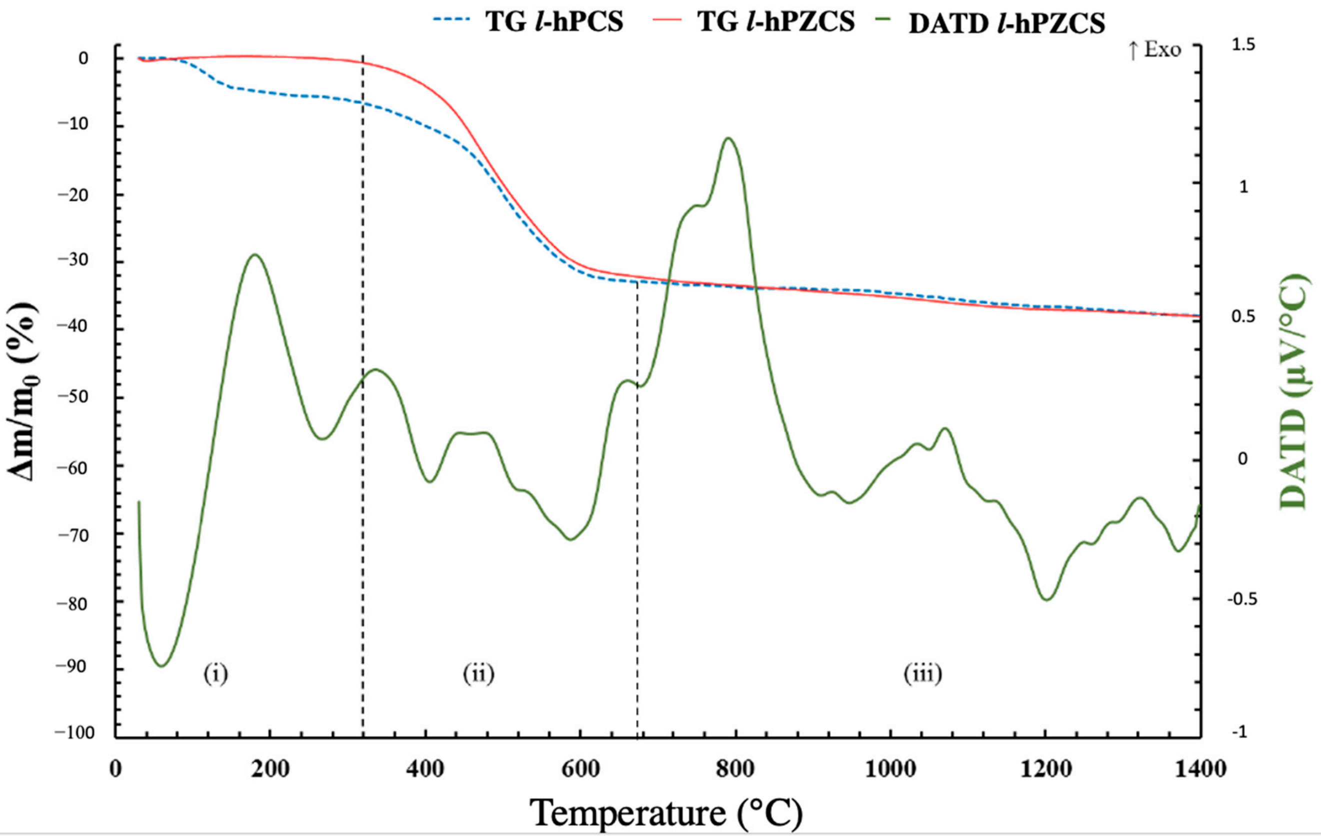

Figure S11. TG profiles of ***l*-hPCS** and ***l*-hPZCS** and DATD of ***hb*-cPZCS** (Exo = exothermic).

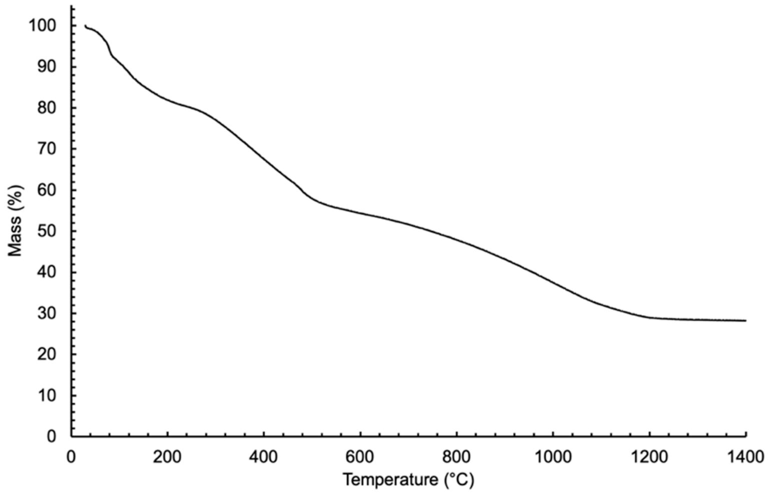

Figure S12. TG profile of $ZrCp_2H_2$.

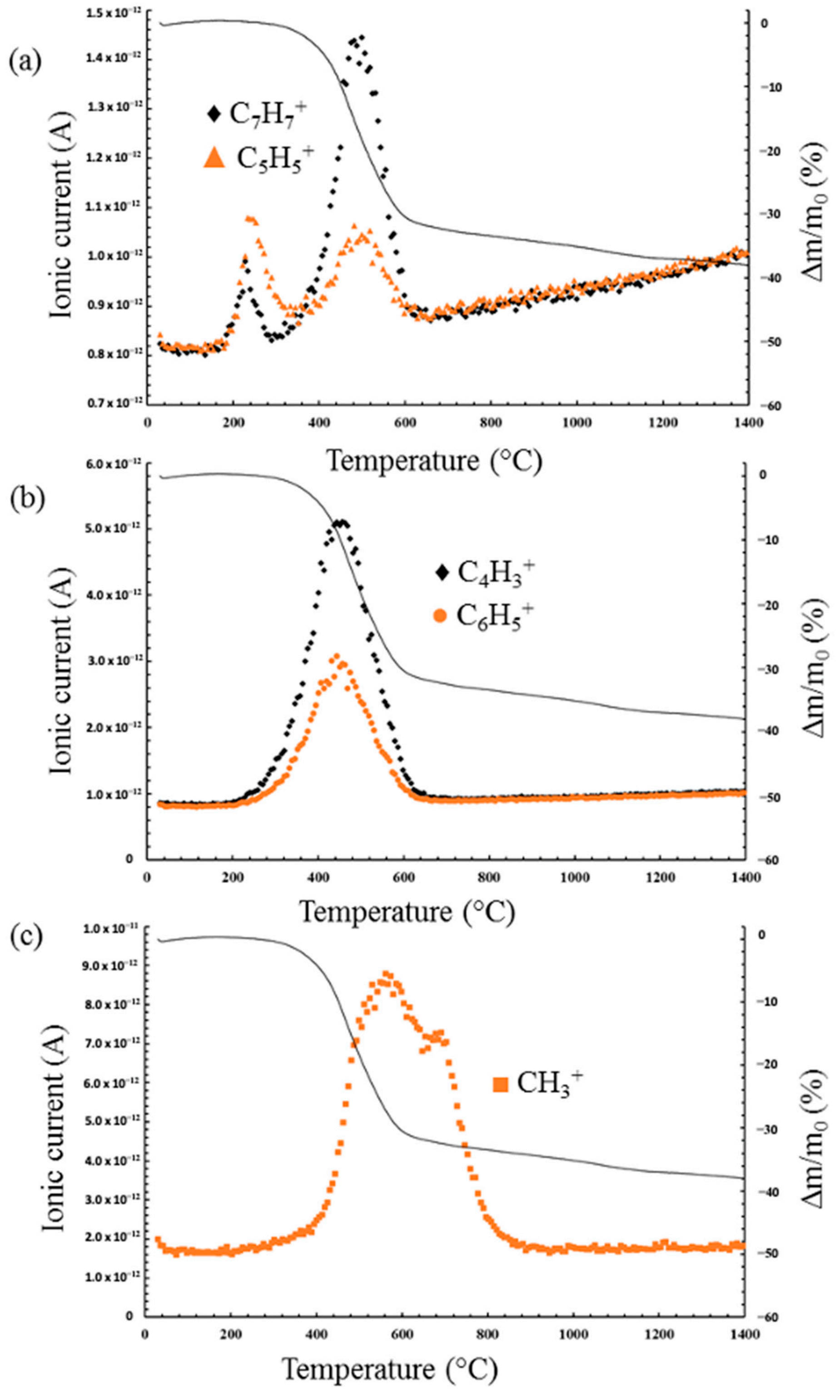

Figure S13. Mass spectrometry of the gaseous species released during the pyrolysis of ***l*-hPZCS**.
